# Snapshot Study on the Value of Omentoplasty in Abdominoperineal Resection with Primary Perineal Closure for Rectal Cancer

**DOI:** 10.1245/s10434-017-6273-9

**Published:** 2017-12-12

**Authors:** Robin D. Blok, Gijsbert D. Musters, Wernard A. A. Borstlap, Christianne J. Buskens, Wilhelmus A. Bemelman, Pieter J. Tanis, Collaborative Dutch Snapshot Research Group, A. Aalbers, Y. Acherman, G. D. Algie, B. Alting von Geusau, F. Amelung, T. S. Aukema, I. S. Bakker, S. A. Bartels, S. Basha, A. J. N. M. Bastiaansen, E. Belgers, W. Bleeker, J. Blok, R. J. I. Bosker, J. W. Bosmans, M. C. Boute, N. D. Bouvy, H. Bouwman, A. Brandt-Kerkhof, D. J. Brinkman, S. Bruin, E. R. J. Bruns, J. P. M. Burbach, J. W. A. Burger, S. Clermonts, P. P. L. O. Coene, C. Compaan, E. C. J. Consten, T. Darbyshire, S. M. L. de Mik, E. J. R. de Graaf, I. de Groot, R. J. L. de Vos tot Nederveen Cappel, J. H. W. de Wilt, J. van der Wolde, F. C. den Boer, J. W. T. Dekker, A. Demirkiran, M. Derkx-Hendriksen, F. R. Dijkstra, P. van Duijvendijk, M. S. Dunker, Q. E. Eijsbouts, H. Fabry, F. Ferenschild, J. W. Foppen, E. J. B. Furnée, M. F. Gerhards, P. Gerven, J. A. H. Gooszen, J. A. Govaert, W. M. U. van Grevenstein, R. Haen, J. J. Harlaar, E. Harst, K. Havenga, J. Heemskerk, J. F. Heeren, B. Heijnen, P. Heres, C. Hoff, W. Hogendoorn, P. Hoogland, A. Huijbers, P. Janssen, A. C. Jongen, F. H. Jonker, E. G. Karthaus, A. Keijzer, J. M. A. Ketel, J. Klaase, F. W. H. Kloppenberg, M. E. Kool, R. Kortekaas, P. M. Kruyt, J. T. Kuiper, B. Lamme, J. F. Lange, T. Lettinga, D. J. Lips, F. Logeman, M. F. Lutke Holzik, E. Madsen, A. Mamound, C. C. Marres, I. Masselink, M. Meerdink, A. G. Menon, J. S. Mieog, D. Mierlo, G. D. Musters, P. A. Neijenhuis, J. Nonner, M. Oostdijk, S. J. Oosterling, P. M. P. Paul, K. C. M. J. C. Peeters, I. T. A. Pereboom, F. Polat, P. Poortman, M. Raber, B. M. M. Reiber, R. J. Renger, C. C. van Rossem, H. J. Rutten, A. Rutten, R. Schaapman, M. Scheer, L. Schoonderwoerd, N. Schouten, A. M. Schreuder, W. H. Schreurs, G. A. Simkens, G. D. Slooter, H. C. E. Sluijmer, N. Smakman, R. Smeenk, H. S. Snijders, D. J. A. Sonneveld, B. Spaansen, E. J. Spillenaar Bilgen, E. Steller, W. H. Steup, C. Steur, E. Stortelder, J. Straatman, M. Stuijvenberg, H. A. Swank, C. Sietses, H. A. ten Berge, H. G. ten Hoeve, W. W. ter Riele, I. M. Thorensen, B. Tip-Pluijm, B. R. Toorenvliet, L. Tseng, J. B. Tuynman, J. van Bastelaar, S. C. van Beek, A. W. H. van de Ven, M. A. J. van de Weijer, C. van den Berg, I. van den Bosch, J. D. W. van der Bilt, S. J. van der Hagen, R. van der Hul, G. van der Schelling, A. van der Spek, N. van der Wielen, E. van Duyn, C. van Eekelen, J. A. Van Essen, K. Van Gangelt, A. A. W. Van Geloven, C. Van Kessel, Y. T. van Loon, A. van Rijswijk, S. J. van Rooijen, T. van Sprundel, L. van Steensel, W. F. van Tets, H. L. van Westreenen, C. J. van de Velde, S. Veltkamp, T. Verhaak, P. M. Verheijen, L. Versluis-Ossenwaarde, S. Vijfhuize, W. J. Vles, S. Voeten, F. J. Vogelaar, W. W. Vrijland, E. Westerduin, M. E. Westerterp, M. Wetzel, M. Wevers, B. Wiering, A. C. Witjes, M. W. Wouters, S. T. K. Yauw, E. C. Zeestraten, D. D. Zimmerman, T. Zwieten

**Affiliations:** 1 Department of Surgery, Academic Medical Centre, University of Amsterdam, Amsterdam, The Netherlands; 2 Centre for Experimental and Molecular Medicine, University of Amsterdam, Amsterdam, The Netherlands

## Abstract

**Background:**

Perineal wound complications are often encountered following abdominoperineal resection (APR). Filling of the pelvic space by omentoplasty (OP) might prevent these complications, but there is scant evidence to support its routine application.

**Objective:**

The aim of this study was to evaluate the impact of OP on perineal wound complications.

**Methods:**

All patients undergoing APR with primary perineal closure (PPC) for non-locally advanced rectal cancer in 71 Dutch centers in 2011 were selected from a cross-sectional snapshot study. Outcomes were compared between PPC with or without OP, which was based on variability in practice among surgeons.

**Results:**

Of 639 patients who underwent APR for rectal cancer, 477 had a non-locally advanced tumor and PPC was performed. Of those, 172 (36%) underwent OP. Patients with OP statistically more often underwent an extralevator approach (32% vs. 14%). Median follow-up was 41 months (interquartile range 22–47). There were no significant differences with or without OP in terms of non-healing of the perineal wound at 30 days (47% vs. 48%), non-healing at the end of follow-up (9% vs. 5%), pelvic abscess (12% vs. 13%) or re-intervention for ileus (5% vs. 3%). Perineal hernia developed significantly more often after OP (13% vs. 7%), also by multivariable analysis (odds ratio 2.61, 95% confidence interval 1.271–5.364; *p* = 0.009).

**Conclusions:**

In contrast to previous assumptions, OP after APR with PPC appeared not to improve perineal wound healing and seemed to increase the occurrence of perineal hernia. These findings question the routine use of OP for primary filling of the pelvic space.

**Electronic supplementary material:**

The online version of this article (10.1245/s10434-017-6273-9) contains supplementary material, which is available to authorized users.

Abdominoperineal resection (APR) is associated with considerable morbidity, particularly regarding the perineal wound.^1^ Reported incidences of perineal wound problems vary widely, but have been observed in up to 47% of patients following APR,^2^ leading to intensive wound care, prolonged hospital stay, and a diminished quality of life. Some patients may experience chronic perineal complications for many years.

As a means of preventing these complications, a variety of techniques using autologous tissue transfer have been proposed. One of the rationales is related to obliterating the pelvic dead space, thereby preventing presacral abscess formation. Furthermore, well-vascularized tissue might have a positive influence on wound healing, especially after radiotherapy. This might reduce perineal wound infection and dehiscence and other complications associated with non-healing. Options for perineal reconstruction following APR include musculocutaneous, fasciocutaneous, subcutaneous, or greater omentum flaps.^3^–^5^ However, the reconstructive procedures are complex, increase operating time, and are associated with a risk of added donor- and recipient-site morbidity (e.g. infection, flap loss). Despite several techniques currently employed for perineal closure after APR, it still remains unclear as to which strategy is superior.

In The Netherlands, the perineal wound after APR for non-locally advanced rectal cancer is most often closed using primary layered suturing of the subcutaneous fat and skin, even in cases of an extralevator approach.

There is no uniformity in the use of omentoplasty (OP), which is performed by approximately one-third of Dutch surgeons. It is hypothesized by surgeons who perform OP that this will improve perineal wound healing and prevent presacral abscess formation by adding well-vascularized and non-irradiated tissue. Although it is primarily intended to obliterate dead space, it has been suggested that an OP might also prevent perineal herniation and small bowel obstruction by preventing descent of bowel loops in the narrow pelvic cavity. Therefore, the aim of this multicenter snapshot study was to evaluate the impact of OP on perineal wound healing, presacral abscess formation, prevention of small bowel obstruction, and development of a perineal hernia in patients undergoing APR with primary perineal wound closure for rectal cancer.

## Patients and Methods

A retrospective cross-sectional snapshot study was performed by the Dutch Snapshot Research Group, evaluating all rectal cancer resections performed in 2011 in 71 hospitals in The Netherlands. This collaborative, resident-led research project has been extensively described in a previous paper from the Dutch Snapshot Research Group.^6^ Using a web-based application, relevant data until the last registered follow-up were collected in 2015. Data entry was performed by one or two residents or research nurses per participating hospital, supervised by a staff member.

Since the present study was retrospectively completed based on electronic patient files with anonymized data analysis, the Medical Ethical Committee of the Academic Medical Centre in Amsterdam, The Netherlands, concluded that written informed consent was not required.

### Patients

Patients were included if the index procedure was an APR for rectal cancer. For the purpose of the present study, only patients in whom primary perineal closure (PPC) was performed, with or without OP, were included for analyses. Patients with locally advanced disease (pT4 stage and/or those who underwent additional visceral resection), autologous tissue flaps, or pelvic floor reconstruction using a mesh were excluded in order to decrease heterogeneity of the cohort and facilitate analysis and interpretation of the data. Patients were also excluded if data on perineal closure were not available.

### Outcome

Primary endpoints were non-healing of the perineal wound at 1, 3, 6, 12 and 18 months and at the end of follow-up, overall incidence of presacral abscess, re-intervention for ileus, and perineal hernia development, irrespective of symptoms. Non-healing of the perineal wound was defined as an open perineal wound. Secondary study endpoints were 30-day mortality, 30-day overall complication rate, need for re-admissions or re-interventions related to the index procedure, local recurrence rate, disease-free survival and overall survival.

### Statistical Analysis

Proportions were expressed as a percentage of the total number of cases in the group. According to distribution, continuous data were reported as mean ± standard deviation (SD) or median with interquartile range (IQR). Numerical data were analyzed using either the *t* test or Mann–Whitney *U* test, while categorical data were analyzed using the *χ* ^2^ test or Fisher’s exact test. Perineal hernia incidence and survival rates were calculated using Kaplan–Meier analysis, and subgroups were compared using the log-rank test. Patients with missing data on perineal hernia status were not included in the Kaplan–Meier analysis. Univariable and multivariable analyses for primary endpoints were performed by binary logistic regression, with separate analyses using Bonferroni correction. Predictors identified in the univariable analysis were candidates for multivariable regression if *p* < 0.2. Significance was set at *p* < 0.05. All analyses were performed using IBM SPSS statistics, version 23.0.0 (IBM Corporation, Armonk, NY, USA).

## Results

### Patient Characteristics

Of the total snapshot cohort of 2102 patients who underwent resection of rectal cancer in 71 Dutch hospitals in 2011, 639 underwent an APR procedure. After excluding locally advanced disease, extended resection and/or additional reconstructive procedures (i.e. flaps/biomesh), a total of 477 patients (172 OP and 305 non-OP) were included in the analyses. Patient baseline characteristics are displayed in Table 1. The mean age was 67 years (± 10.8) and 70% were male. A total of 96% of patients received neoadjuvant radiotherapy and 8% received adjuvant chemotherapy, with no significant differences between the groups. The proportion of OP at hospital level was 0% in 17 hospitals (27%), between 1 and 25% in 14 hospitals (22%), between 26 and 75% in 18 hospitals (29%), and between 76 and 100% in 14 hospitals (22%). Patients who underwent OP more often had diabetes mellitus, a higher percentage underwent extralevator APR, and a laparoscopic approach was used less often (Table 1). Total follow-up after OP was 42 months (IQR 25–46), which was similar to a follow-up of 40 months (IQR 22–47) in the non-OP group.

**Table 1:** Baseline characteristics

	Group A	Group B	
	No omentoplasty [*n* = 305]	Omentoplasty (*n* = 172)	*p* value
Hospital			
Non-teaching hospital	59 (19)	26 (15)	0.001
Teaching hospital	231 (76)	120 (70)	
University hospital	15 (5)	26 (15)	
Sex			
Male	203 (67)	129 (75)	0.054
Age, years			
Mean ± SD	68 ± 11	66 ± 11	0.131
Body mass index, kg/m^2^			
Mean ± SD	26 ± 4	27 ± 4	0.007
ASA classification			
1	86 (29)	41 (24)	0.526
2	166 (55)	100 (59)	
3	47 (16)	26 (15)	
4	1 (0.3)	2 (1.2)	
Comorbidity			
Diabetes mellitus	25 (8)	32 (19)	0.001
Vascular	110 (37)	57 (34)	0.540
Previous operations			
Abdominal surgery	101 (34)	51 (30)	0.453
Pelvic surgery	34 (12)	16 (10)	0.530
Distance to anorectal junction, cm			
Median (IQR)	2 (1–4)	2 (0–4)	0.782
Relation tumor to MRF on MRI			
< 1 mm	107 (42)	75 (53)	0.035
Neoadjuvant radiotherapy			
Total	278 (96)	150 (94)	0.363
Surgery prior to resection			
Stoma	22 (7)	7 (4)	0.168
Type of surgery			
iAPR	23 (8)	11 (7)	0.641
cAPR	227 (78)	100 (61)	< 0.001
eAPR	41 (14)	53 (32)	< 0.001
Approach			
Laparoscopic	164 (55)	56 (33)	< 0.001
Conversion			
Total	16 (10)	3 (6)	0.288
Intraoperative complication^a^			
Total	15 (5)	10 (6)	0.686
Adjuvant chemotherapy			
Total	25 (8)	15 (9)	0.859

Data are expressed as *n* (%) unless otherwise specified

*ASA* American Society of Anesthesiologists, *MRF* mesorectal fascia, *MRI* magnetic resonance imaging, *i/c/eAPR* intersphincteric/conventional/extralevator abdominoperineal resection, *SD* standard deviation, *IQR* interquartile range

^a^Includes injury to spleen, intestine, ureter/urethra, bladder and vagina, and bleeding for which transfusion was required

### Primary Endpoints

**Fig. 1:**
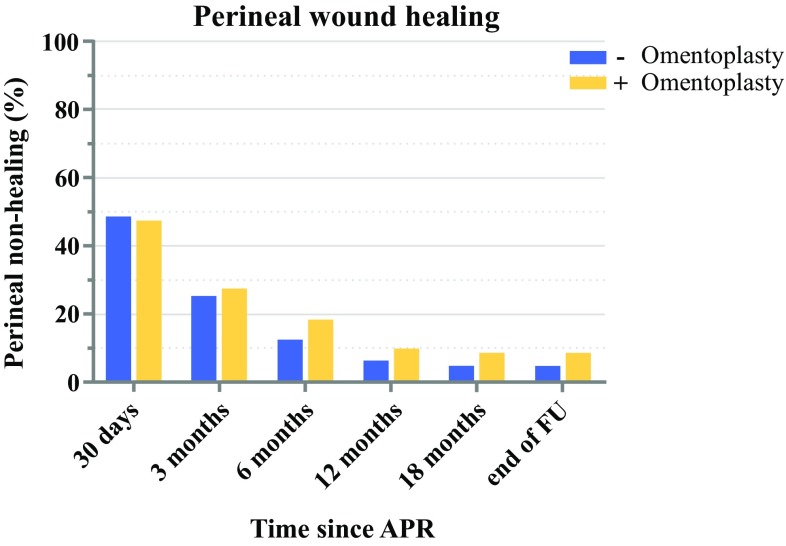
Perineal wound healing over time with and without omentoplasty. *APR* abdominoperineal resection, *FU* follow-up

There were no significant differences between groups in proportions with a primary healed perineal wound, at any time point during follow-up (Fig. 1). After 30 days, the non-healing rate was 47% (72/152) after OP and 48% (132/272) without OP (*p* = 0.819). At the end of follow-up, the rate of chronic non-healing of the perineal wound was 9% (13/152) and 5% (13/272) in the OP and non-OP groups, respectively (*p* = 0.120). By univariable logistic regression, the following variables reached *p* < 0.2: age, diabetes mellitus, OP, and type of APR (electronic supplementary Table S1). Using a multivariable model, non-healing of the perineal wound at 30 days and 3 months postoperatively occurred significantly less often after an intersphincteric approach if compared with a conventional APR (Table 2). Age was also independently associated with non-healing at 30 days, with an OR of 1.03 [95% confidence interval (CI) 1.010–1.049] for increasing age of 1 year each (*p* = 0.003).

**Table 2:** Multivariable logistic regression analysis

Parameter	Perineal hernia		Open wound, 30 days		Open wound, 3 months		Open wound, 12 months		Open wound, end of follow-up	
	OR	*p* value	OR	*p* value	OR	*p* value	OR	*p* value	OR	*p* value
Omentoplasty	2.61	0.009	NI	NI	NI	NI	1.46	0.324	1.59	0.279
eAPR^a^	NI	NI	1.03	0.910	1.06	0.846	NI	NI	NI	NI
iAPR^a^	NI	NI	0.40	0.024	0.25	0.027	NI	NI	NI	NI
Open approach^b^	NI	NI	–	–	–	–	–	–	–	–
Female sex	2.42	0.021	NI	NI	NI	NI	NI	NI	NI	NI
Age^c^	NI	NI	1.03	0.003	1.02	0.093	1.02	0.192	1.03	0.181
Diabetes mellitus	–	–	NI	NI	NI	NI	1.64	0.319	2.14	0.139
Vascular disease	–	–	NI	NI	NI	NI	NI	NI	NI	NI
Previous pelvic surgery^d^	1.18	0.760	–	–	–	–	–	–	–	–
Neoadjuvant radiotherapy	0.29	0.038	NI	NI	NI	NI	NI	NI	NI	NI

*OR* odds ratio, *APR* abdominoperineal resection, *e/iAPR* extralevator/intersphincteric abdominoperineal resection, *NI* not included based on univariate analysis

^a^Conventional APR as a reference

^b^Compared with the transabdominal laparoscopic procedure

^c^Included as a continuous variable

^d^Includes hysterectomy, prostatectomy, cystectomy, and ovariectomy

During complete follow-up, a presacral abscess developed in 12% (21/170) of patients after OP, which did not significantly differ from the 13% (39/300) of patients without OP (*p* = 0.840). Univariable logistic regression analysis showed no significant influence of baseline characteristics on abscess formation (electronic supplementary Table S1).

The re-admission rate for ileus was 5% (8/172) in the OP group and 7% (21/305) in the non-OP group (*p* = 0.327). The re-intervention rate pertaining to small bowel obstruction was also equivalent between groups [5% (8/172) OP vs. 3% (9/305) non-OP; *p* = 0.336].

**Fig. 2:**
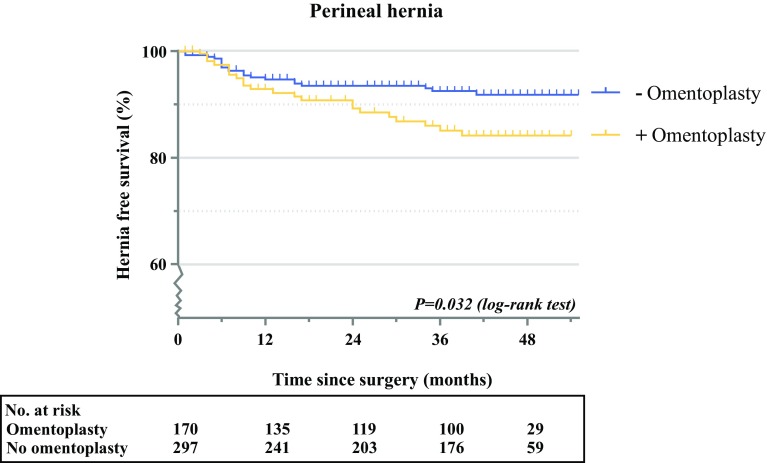
Kaplan–Meier curve for developing a perineal hernia over time

The median duration between APR and perineal hernia development was 9 months (IQR 6–21). Perineal herniation occurred significantly more often after APR with OP compared with APR without OP (13% vs. 7%; OR 2.61, 95% CI 1.271–5.364; *p* = 0.009) [Table 2, and electronic supplementary Table S1], and also after Bonferroni correction (electronic supplementary Table S3). Over time, perineal herniation appeared to stabilize in the non-OP group after 24 months, while perineal hernias continued to occur beyond 2 years in the OP group (Fig. 2; *p* = 0.032 [log-rank test]). Females also had an increased risk of developing a perineal hernia after APR compared with men (OR 2.42, 95% CI 1.141–5.135; *p* = 0.021). Neoadjuvant radiotherapy was associated with a lower risk of perineal herniation (OR 0.29, 95% CI 0.088–0.934; *p* = 0.038). An extralevator approach was not significantly associated with perineal hernia development in univariable analysis (*p* > 0.2) and was therefore not included in the multivariable model (Table 2).

### Secondary Endpoints

At 30 days postoperatively, the overall complication rate was 37% (174/465). Surgical complications, re-intervention for a surgical complication, and 30-day mortality rate did not significantly differ between groups (electronic supplementary Table S2). In the period after 30 days postoperatively, the re-admission rate was not decreased by use of an OP compared with no OP (20% vs. 23%, respectively; *p* = 0.499), and neither was the need for reoperations (16% vs. 13%, respectively; *p* = 0.329). Three-year local recurrence (6% vs. 3%), disease-free survival (66% vs. 67%), and overall survival (80% vs. 80%) did not differ between groups (electronic supplementary Table S4).

## Discussion

This snapshot study is the largest reported comparative cohort study to date on the effect of OP on perineal wound complications after APR in a homogenous patient population. Performing an OP in combination with primary wound closure appeared not to improve perineal wound healing. Filling of the pelvic dead space by OP was also not associated with fewer pelvic abscesses or re-interventions for ileus. Moreover, OP was an independent risk factor for perineal hernia formation, besides female sex.

**Fig. 3:**
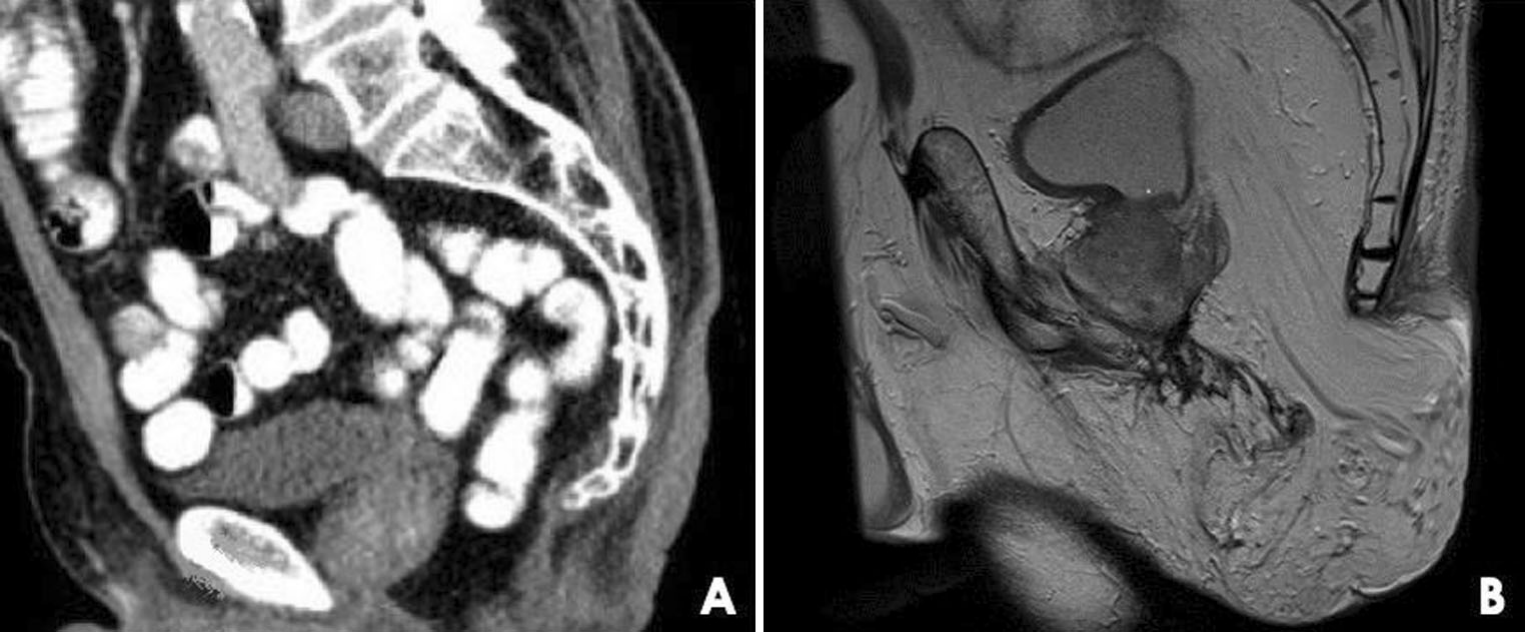
Two male patients after abdominoperineal resection with primary perineal closure for rectal cancer. **a** Computed tomography image, sagittal plane, showing descent of a small bowel loop with restricted mesenteric length. **b** Magnetic resonance image, sagittal plane, showing a large perineal hernia in which the hernia sac is filled with fully mobilized bulky omentum

Despite its frequent use and clinical implications, there are only very limited data on OP for filling of the pelvic cavity after APR. A counterintuitive finding was the higher perineal hernia rate after OP, while the extralevator approach was not associated with perineal hernia development. The incidence of perineal hernia was based on documentation in the patient files, without predefined definition. The 13 and 7% hernia rates in the two groups are higher than the rates mostly reported in the literature, but are even likely to be underestimated (small asymptomatic hernias are probably not documented). An OP is often considered to be a perineal reconstruction technique, but the omental fat is actually frequently the content of the hernia sac if a perineal hernia occurs. A plausible explanation may be that a fully mobilized OP with a long vascular pedicle allows for more herniation than descending small bowel loops that are restricted by mesenteric length (Fig. 3). After removal of the rectum, the bladder and internal genital organs move posteriorly and reduce the presacral space towards the pelvic outlet. This sometimes even prevents the small bowel to fully descend to the closed perineum, or it is only a single loop that fills the presacral space. In contrast, an OP will prevent the bladder and internal genital organs from displacing and a large bulk of omental fat will give downward pressure on the perineal wound in a standing position. That the extralevator approach was not associated with perineal hernia formation might be explained by the fact that surgeons performing a ‘conventional’ APR have already adopted elements of cylindrical resection without changing the name of their surgical technique. The protective effect of radiotherapy for developing a perineal hernia was also remarkable, but might be explained by inducing fibrosis,^7^ which in turn might strengthen the perineal scar. However, these results should be interpreted with caution since 96% of patients received radiotherapy.

It has been proposed that an OP would lower infectious complications after APR by obliterating the perineal dead space, which reduces the formation of fluid collections with secondary infection. Furthermore, OP might promote angiogenesis and might enhance local immunity and antibiotic delivery.^8^–^10^ Given the often irradiated fibrotic pelvic tissues, adding well-vascularized tissue might enhance the local wound healing. Besides its potential role in reducing infectious complications, it has been suggested that OP might prevent small bowel descent with the risk of ileus. However, none of these potential advantages of OP could be demonstrated in the present study.

In a randomized controlled trial on biomesh repair after APR, we have already demonstrated that OP did not improve wound healing, based on a post hoc analysis.^11^ OP was performed in 61 of the 101 included patients, with no impact of OP on perineal wound complications (RR 1.111, 95% CI 0.651–1.897). This snapshot study confirms this observation in a large observational cohort. Killeen et al. published a systematic review in which they included all APR cohort series mentioning the use of OP, regardless of indication.^12^ They found an improved primary wound healing rate, more rapid healing, and fewer infectious complications after OP. These contradictory findings, compared with the present study, are likely to be related to several methodological shortcomings of the studies included in the review. These consisted of heterogeneous and mostly historical patient populations with confounded comparisons. Half of the studies did not include a control, with only three small comparative series since 2000. Furthermore, data were pooled from studies containing inflammatory bowel disease together with series only describing cancers. Cancer patients received neoadjuvant radiotherapy in a wide range, between 14 and 75%, which is one of the major determinants for perineal wound complications. The extent of the resection and use of myocutaneous flaps was not evenly distributed among groups, without the ability to correct for these confounders. Finally, the pooled median follow-up was only 13.5 months, with merely two studies exceeding 24 months. This underlines the importance of the present study in which the impact of OP is evaluated in a large homogeneous patient cohort with a median follow-up of 41 months.

A possible explanation for not finding an impact of OP on abscess formation and perineal wound healing may be related to insufficient bulk of tissue because of low body mass index or inadequate mobilization. There is no consensus on the technical aspects of detachment of the omentum, whether to create a vascular pedicle on the right or left gastroepiploic artery, and the route along which the OP is positioned in the pelvic cavity (i.e. left paracolic gutter or via a mesenteric window). An insufficient OP leads to a small residual cavity, providing an opportunity for abscess formation. Another possibility is that the larger perineal defects were selected for OP, thus leading to an underestimation of the added effect of OP in the prevention of perineal wound complications. However, the observed perineal non-healing and abscess rates do not indicate any trend favoring OP, and extensive resections for locally advanced disease were excluded.

There are some limitations to this study, inherent to its retrospective and non-randomized design. Selection of participating centers could have introduced bias; however, the study included a relatively large number of hospitals and cases of APR. We also recognize limitations due to the unavailability of some relevant variables such as indication for OP, technical details of the OP, i.e. type of vascular pedicle, postoperative drain use, or the extent of the perineal wound, and technical details on perineal closure (e.g. layered suturing, leaving the skin open). Finally, the results might have been biased by allocation. However, the divergent proportions of OP applied per center indicate that the decision of using OP was mainly surgeon- or hospital-related, rather than patient-related.

Although the physiological properties of the omentum make it an excellent hypothetical candidate for routine use following APR, the present study found no evidence to support an OP for improvement of perineal wound healing or reducing the risk of postoperative ileus. On the contrary, OP seemed to be associated with a higher incidence of perineal hernia. Furthermore, OP results in a longer operating time and has a reported risk of necrosis and prolonged postoperative ileus, although incidences seem to be low.^12^, ^13^ The only potential improvement by performing an OP might be regarding preservation of bladder and sexual function by preventing posterior displacement, which was not investigated in the present study. Considering the implications for current daily practice, the present study, as well as the low-quality available literature, do not support routine use of an OP.

## Conclusions

In the absence of randomized controlled trials, this large, comparative cohort study provides the best available evidence on the additional value of OP in patients undergoing APR for rectal cancer with PPC and almost routine use of radiotherapy. OP did not have any impact on abscess formation, postoperative ileus, or (time to) perineal wound healing.

## Electronic Supplementary Material

Below is the link to the electronic supplementary material.

Supplementary material 1 (DOC 118 kb)

Supplementary material 2 (XLS 47 kb)
