# Supplementary material for: Snapshot Study on the Value of Omentoplasty in Abdominoperineal Resection with Primary Perineal Closure for Rectal Cancer
Source: Ann Surg Oncol. 2017 Dec 12;25(3):729–36. doi: 10.1245/s10434-017-6273-9 (PMC5814519; doi:10.1245/s10434-017-6273-9)
Supplement: Supplementary file 1 — Supplementary material 1 (DOC 118 kb) [file 10434_2017_6273_MOESM1_ESM.doc]

| **Supplement table 1. Univariable logistic regression analysis** | | | | | | | |  |  |  |  |  |  |  |  |  |  |  |
| --- | --- | --- | --- | --- | --- | --- | --- | --- | --- | --- | --- | --- | --- | --- | --- | --- | --- | --- |
|  |  | **Abscess** |  |  | **Perineal hernia** | |  | **Open wound 30d** | |  | **Open wound 3m** | |  | **Open wound 12m** | |  | **Open wound eFU^a^** | |
| **Parameter** |  | Odds ratio | *p-value* |  | *Odds ratio* | *p-value* |  | *Odds ratio* | *p-value* |  | *Odds ratio* | *p-value* |  | *Odds ratio* | *p-value* |  | *Odds ratio* | *p-value* |
| Omentoplasty |  | 0.94 | 0.840 |  | 2.05 | 0.027 |  | 0.96 | 0.819 |  | 1.12 | 0.611 |  | 1.64 | 0.180 |  | 1.86 | 0.125 |
| eAPR^b^ |  | 1.49 | 0.232 |  | 1.20 | 0.660 |  | 0.92 | 0.747 |  | 0.99 | 0.984 |  | 0.54 | 0.267 |  | 0.68 | 0.496 |
| iAPR^b^ |  | 0.73 | 0.614 |  | 1.88 | 0.230 |  | 0.43 | 0.034 |  | 0.27 | 0.033 |  | 0.33 | 0.279 |  | <0.001 | 0.998 |
| Open approach^c^ |  | - | - |  | 0.87 | 0.675 |  | - | - |  | - | - |  | - | - |  | - | - |
| Gender *(female)* |  | 0.82 | 0.522 |  | 2.08 | 0.026 |  | 0.94 | 0.772 |  | 0.84 | 0.461 |  | 0.94 | 0.871 |  | 1.32 | 0.508 |
| Age^d^ |  | 0.99 | 0.259 |  | 1.01 | 0.389 |  | 1.03 | 0.009 |  | 1.01 | 0.183 |  | 1.03 | 0.159 |  | 1.03 | 0.134 |
| Diabetes Mellitus |  | 1.82 | 0.104 |  | - | - |  | 1.43 | 0.245 |  | 1.47 | 0.243 |  | 1.97 | 0.160 |  | 2.64 | 0.050 |
| Vascular disease |  | 1.01 | 0.976 |  | - | - |  | 1.12 | 0.575 |  | 0.97 | 0.899 |  | 1.24 | 0.577 |  | 1.56 | 0.286 |
| Previous pelvic surgery^e^ |  | 0.94 | 0.899 |  | 1.96 | 0.133 |  | - | - |  | - | - |  | - | - |  | - | - |
| Neoadjuvant radiotherapy |  | 0.79 | 0.720 |  | 0.34 | 0.068 |  | 1.18 | 0.752 |  | 1.51 | 0.526 |  | 1.14 | 0.902 |  | 0.91 | 0.932 |
| *^a^end of follow-up; ^b^conventional APR as a reference; ^c^compared to transabdominal laparoscopic procedure; ^d^included as a continous variable; ^e^includes hysterectomy, prostatectomy, cystectomy and ovariectomy.* | | | | | | | | | | | | | | | | | | |
|
|

| **Supplement table 2. Postoperative outcome within 30 days after APR** | | | |  |  |
| --- | --- | --- | --- | --- | --- |
|  |  | **Group A** |  | **Group B** |  |
|  |  | *No omental plasty* |  | *Omental plasty* | *p-value* |
|  |  | (n=305) |  | (n=172) |  |
| Surgical complications | *Overall, n (%)* | 64 (22) |  | 33 (20) | 0.621 |
| Non-surgical complications | *Overall, n (%)* | 39 (13) |  | 32 (19) | 0.089 |
| Reintervention^a^ | *Total, n (%)* | 35 (12) |  | 18 (11) | 0.885 |
|  | *Abscess drainage, n (%)* | 11 (4) |  | 6 (4) | 0.928 |
|  | *Ileus, n (%)* | 3 (1) |  | 0 (0) | 0.557 |
|  | *Bleed, n (%)* | 5 (2) |  | 4 (2) | 0.729 |
|  | *Other, n (%)* | 14 (5) |  | 8 (5) | 0.997 |
| Approach reintervention | *Open procedure, n (%)* | 17 (6) |  | 12 (7) | 0.546 |
| Mortality | *Total, n (%)* | 7 (2) |  | 7 (4) | 0.257 |
| *^a^For perineal wound problems* | | | | | |

| **Supplement table 3.** | |  |  |  |  |
| --- | --- | --- | --- | --- | --- |
| **Impact of omentoplasty on perineal hernia after Bonferroni correction** | | | | | |
|  |  | **Perineal hernia** | |  |  |
| **Parameter** | | *Odds*  *ratio* | *p-value* |  | *95% C.I.^a^* |
| **Omentoplasty** | *Unadjusted* | 2.05 | 0.027 |  | 1.084 - 3.881 |
|  |  |  |  |  |  |
|  | *Minmally adjusted^b^* | 2.57 | 0.024 |  | 1.273 - 5.180 |
|  |  |  |  |  |  |
|  | *Maximally adjusted^c^* | 2.61 | 0.036 |  | 1.271 - 5.364 |
|  |  |  |  |  |  |
| *^a^Confidence interval; ^b^adjusted for type of abdominoperineal resection and neoadjuvant radiotherapy; ^c^adjusted for gender, neoadjuvant radiotherapy and previous pelvic surgery based on univariable regression.* | | | | | |
|
|
|

| **Supplement table 4. Oncological outcome at 3 years postoperatively** | | | | | | | |
| --- | --- | --- | --- | --- | --- | --- | --- |
|  |  |  | **Group A** |  |  | **Group B** |  |
|  |  | *No omental plasty* | | |  | *Omental plasty* | *p-value* |
|  |  |  | (n=305) |  |  | (n=172) |  |
| Radical resection | *CRM >1mm, n (%)* |  | 215 (90) |  |  | 133 (91) | 0.804 |
| ypTNM-stage | *Stage I, n (%)* |  | 104 (39) |  |  | 63 (41) | 0.337 |
|  | *Stage II, n (%)* |  | 64 (24) |  |  | 27 (17) |
|  | *Stage III, n (%)* |  | 76 (29) |  |  | 46 (30) |
|  | *Stage IV, n (%)* |  | 23 (9) |  |  | 19 (12) |
| Local recurrence | *Total, n (%)* |  | 10 (3) |  |  | 11 (6) | 0.111 |
| Time to recurrence | *Months, median (IQR)* | | 23 (17-32) |  |  | 16 (8-25) | 0.148 |
| Overall survival | *Total, n (%)* |  | 245 (80) |  |  | 137 (80) | 0.859 |
| Disease free survival | *Total, n (%)* |  | 205 (67) |  |  | 113 (66) | 0.736 |
| *CRM=circumferential resection margin; ypTNM=pathological tumour staging after neoadjuvant therapy.* | | | | | | | |
|
